# Improved staging using intraoperative ultrasound for mediastinal lymphadenectomy in non-small lung cancer surgery

**DOI:** 10.1186/1749-8090-8-S1-O225

**Published:** 2013-09-11

**Authors:** N Ilic, J Juricic, J Banovic, D Krnic, O Barcot

**Affiliations:** 1 Thoracic Surgery Dept., University Surgical Hospital, Split, Croatia

## Objectives

The extend of lymph node involvement in patients NSCLC is the cornerstone of staging and influences both multimodality treatment and final outcome. We studied safety, accuracy and characteristics of intraoperative ultrasound guided systematic mediastinal nodal dissection in patients with resected NSCLC.

## Methods

Prospective randomized trial of intraoperative surgical staging after radical surgery for NSCLC was carried out. Intraoperative hand held ultrasound probe was used in systematic mediastinal nodal dissection in 124 patients after radical surgery for NSCLC and compared with 120 patients who underwent radical surgery followed by standard systematic mediastinal nodal dissection. Mapping of the lymph nodes by their number and sation followed by histopathologic evaluation was performed. Patients data were statistically analyzed.

## Results

The surgical procedure used was comparable in both groups of patients. Operating time was prolonged for 10 ( 6-20 ) minutes in patients with US guided mediastinal nodal dissection, but number and stations of evaluated lymph nodes were significantly higher ( p<0.001 ) at the same group of patients. Skip nodal metasteses were found in 24 % of patients without N1 nodal involvement. We upstaged 12 ( 10 % ) patients using US guided mediastinal lyphadenectomy. Median follow-up was 38 ( range 10-52 ) months. Standard staging system seemed to be improved in US guided mediastinal lyphadenecetomy patients. Complication rate showed no difference between analyzed groups of patients.

## Conclusion

Higher number and location of analyzed mediastinal nodal stations in patients with resected NSCLC using hand held ultrasound probe siggested to be of great oncological significance. Procedure showed absolute safety and high accuracy. Our results indicate that intraoperative US may have important staging implication. Further clinical studies should be performed in order to improve intraoperative staging in NSCLC patients.

